# Socioeconomic Deprivation and Vocal Handicap in Adults With Voice Disorders

**DOI:** 10.1002/lary.70510

**Published:** 2026-03-23

**Authors:** Robert Brinton Fujiki, Susan L. Thibeault

**Affiliations:** ^1^ Department of Otolaryngology—Head & Neck Surgery Indiana University School of Medicine Indianapolis Indiana USA; ^2^ Department of Otolaryngology—Head & Neck Surgery University of Wisconsin‐Madison Madison Wisconsin USA

**Keywords:** dysphonia, quality of life, socioeconomic status, voice disorders, Voice Handicap Index

## Abstract

**Objective:**

This study examined the relationship between socioeconomic deprivation and quality of life as measured by the Voice Handicap Index (VHI) in adults with voice disorders.

**Methods:**

A cross sectional design was utilized. One‐thousand one‐hundred and twenty adults with voice disorders were included in this study (mean age = 53.6, SD = 16.5; males = 429 females = 788). Patients were divided into the following diagnostic groups: muscle tension dysphonia (32%), benign vocal fold lesions (29.4%), laryngospasm (12.3%), vocal fold paralysis (11.1%), neurological voice disorders (6.2%), laryngeal cancer (5.9%), and presbyphonia (3%). VHI scores, Grade Roughness Breathiness Asthenia and Strain (GRBAS) ratings, age, sex, employment status, and smoking history were extracted from initial voice evaluations. Socioeconomic deprivation was measured using the Area Deprivation Index (ADI).

**Results:**

Patients living in areas with the highest socioeconomic deprivation presented with greater total (*p* < 0.001), functional (*p* < 0.001), physical (*p* < 0.001), and emotional VHI scores (*p* < 0.001) when compared to those living in more affluent areas—even when controlling for dysphonia severity, age, sex, voice‐related diagnosis, employment status, and smoking history. The association between VHI and ADI was strongest for total score and emotional subscore, as VHI total scores increased by 0.32 (95% CI = 0.26–1.26, *p* < 0.001) and VHI Emotional scores increased by 0.18 (95% CI = 0.16–0.21, *p* < 0.001) for every 1‐point increase in ADI.

**Conclusions:**

Patients living with socioeconomic deprivation experienced greater voice‐related handicap from voice disorders than those from more affluent backgrounds. Future work is needed to better characterize the relationship between quality of life and socioeconomic deprivation in those with voice disorders.

**Level of Evidence:**

3.

## Graphical abstract

This study examined the relationship between socioeconomic deprivation and quality of life as measured by the Voice Handicap Index (VHI) in adults with voice disorders. Patients living with socioeconomic deprivation experienced greater voice‐related handicap from voice disorders than those from more affluent backgrounds, even when controlling for dysphonia severity, voice‐related diagnosis, and patient demographics. Future work is needed to better characterize the relationship between quality of life and socioeconomic deprivation in those with voice disorders.

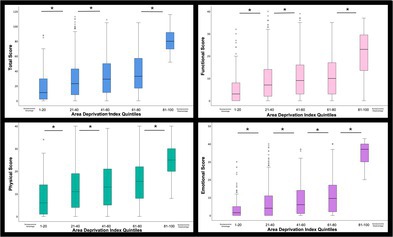

## 1 Introduction

Voice disorders disrupt effective communication and undermine quality of life [1] for as many as 6% of adults and children in the United States (US) [2, 3, 4, 5, 6, 7]. Estimates of lifetime prevalence for voice disorders range from 20% to 33% [3, 4, 5, 8]. The influence of voice problems can be wide reaching—affecting both social [9, 10] and occupational function [11, 12, 13]. In addition, an individual's voice is connected to their sense of identity [14], and the emotional sequalae associated with dysphonia can be significant [1, 15, 16]. Unfortunately, audio‐perceptual and quantitative measures of voice function are not designed to consider the influence of dysphonia on an individual's self‐image or quality of life.

In order to assess the social and emotional impact of voice disorders, a variety of patient‐reported measures have been developed [17]. The Voice Handicap Index (VHI) [18] is a reliable tool [19] that separates individuals with and without voice complaints [20, 21, 22]. The VHI assesses overall voice‐related handicap as well as the emotional, physical, and functional influence of dysphonia [18]. This measure has been shown to be sensitive to the effects of voice treatment [23], and is commonly employed to probe the manner in which vocal function limits quality of life [24, 25].

In order to describe how voice disorders influence quality of life, researchers have examined factors associated with VHI outcomes [26, 27, 28, 29]. For example, voice quality as measured by the Grade Roughness Breathiness Asthenia and Strain (GRBAS) scale predicts overall VHI score [26, 30]. Noise to Harmonic Ratio and fundamental frequency weakly predict VHI outcomes [26, 27], as does smoking status [27]. These findings suggest that voice disorder severity influences patients' life experience. However, many, if not most, of the factors driving VHI scores are not assessed in typical voice evaluations.

As one example, socioeconomic status (SES) is a potentially important factor influencing the impact of a voice disorder on a patient's life [22, 31]. In fact, the prevalence of voice disorders may be inversely related to income [22, 32]. Triage patterns may differ across patients living in different geographical areas (i.e., metropolitan vs. urban) [33], and lower income patients are more likely to postpone voice‐related treatments for longer periods of time following symptom onset [32]. When treatment is initiated, patients with lower educational levels experience poorer improvements in VHI scores 6 months following treatment [28].

Low SES negatively influences a variety of health‐related outcomes across medical specialties [34]. It is the case, however, that SES is a multifactorial and complex construct, and investigating the relationship between SES and voice disorders is challenging [35]. SES involves an individual's educational, employment, economic, housing, and social opportunities [36], and thus, it can be complex to quantify. For this reason, SES is sometimes examined on a neighborhood level [37, 38, 39, 40]. The Area Deprivation Index (ADI) has been developed as a tool to account for the multitude of factors which encompass socioeconomic deprivation [41]. This methodology has potential for characterizing the manner in which socioeconomic deprivation influences the relationship between voice disorders and quality of life.

This study investigated the manner in which socioeconomic deprivation influences VHI outcomes in adult patients with voice disorders. VHI scores from baseline voice evaluations were compared across patients living in areas of varying socioeconomic deprivation as measured by the ADI. Voice quality severity, age, sex, employment status, voice‐related diagnosis, and smoking status were accounted for across all patients. It was hypothesized that when compared with patients from more affluent areas, patients living in areas with greater socioeconomic deprivation might experience greater voice‐related handicap.

## 2 Methods

### 2.1 Study Design

A cross‐sectional design was utilized. VHI outcomes were compared across patients with voice disorders living in areas with varying socioeconomic deprivation as measured by the Area Deprivation Index (ADI).

### 2.2 Population

Data were collected from the University of Wisconsin‐Madison Voice and Swallow Clinics Outcome Database. This database is managed by the University of Wisconsin‐Madison School of Public Health and Institutional Review Board. When patients presented to the Voice and Swallow Clinic, they were consented for database inclusion. At the time of this investigation, the database contained 6838 patients representing a myriad of diagnoses. For study inclusion, patients were required to (A) have undergone an evaluation for a primary voice complaint with a certified laryngologist and a voice‐specialized speech language pathologist (SLP), (B) be > 18 years of age, (C) live in a residence with a 9‐digit zip code, and (D) have completed the VHI during their visit. Patients in this study presented to the clinic between April 2009 and June 2021. Data were collected from initial voice evaluations.

### 2.3 Demographics

Age in years, sex on birth certificate, presence of smoking history (yes/no), and number of years smoked were extracted. Additionally, employment status was collected and was categorized as either working fulltime, working part‐time, retired, on disability, or unemployed.

### 2.4 Voice Related Diagnosis

Voice‐related diagnoses as determined by laryngologists were collected. Patient diagnoses were divided into seven categories including (1) muscle tension dysphonia, (2) vocal fold paralysis or paresis, (3) benign vocal fold lesions, (4) neurological voice disorders (i.e., spasmodic dysphonia), (5) laryngospasm, (6) laryngeal cancer or recurrent respiratory papilloma, (7) age related glottic insufficiency (henceforth referred to as presbyphonia) [42]. To receive a diagnosis of muscle tension dysphonia, patients were required to present with dysphonia in the absence of an organic or neurological etiology [43, 44]. Common videostroboscopic findings for this diagnosis were defined based on past study and included laryngeal hyperfunction, supraglottic constriction, and/or vocal fold hypoadduction during vocalization [44]. Patients diagnosed with laryngospasm presented to the clinic for a voice complaint in the presence of dyspnea symptoms and the absence of other laryngeal abnormalities.

### 2.5 Auditory‐Perceptual Voice Assessment

For each patient, voice quality was rated using the Grade Roughness Breathiness Asthenia, Strain (GRBAS) scale [45]. Ratings were made by SLPs specializing in voice. All clinicians were trained on auditory‐perceptual voice assessments upon hire and completed intermittent consensus trainings to ensure intra and inter‐rater reliability [46]. All clinicians had a minimum of 5 years' experience treating voice or were clinical fellows working under the supervision of a licensed SLP.

### 2.6 Voice Handicap Index (VHI)

Patients completed the VHI during their evaluations, and responses were extracted from the database. Total scores, as well as functional, physical, and emotional subscores, were then calculated following the published VHI protocol [18].

### 2.7 Area Deprivation Index (ADI)

Patient 9‐digit‐zip codes were converted into ADI scores. ADI quantifies socioeconomic deprivation on a neighborhood level (census block group) [41]. ADI scores consider neighborhood income, employment, education, and quality of housing [41]. National ADI scores rank neighborhoods from 1 to 100 (1 = greatest advantage, 100 = greatest disadvantage) in reference to the overall country [41, 47].

#### 2.7.1 Statistical Analysis

Descriptive statistics were performed. Linear regression was used to test the relationship between VHI outcomes and ADI scores. To account for other factors which may drive VHI scores, GRBAS score (overall grade of dysphonia), age, sex, employment status, smoking history (yes/no), and voice‐related diagnosis were included in the regression model. Tukey's pairwise tests were used for post hoc analyses. For post hoc analyses, ADI scores were divided into quintiles (1–20, 21–40, 41–60, 61–80, 81–100). In order to protect against type 1 error, alpha was set at 0.01 for determining statistical significance. Analyses were performed using SAS (Version 26).

## 3 Results

One‐thousand one‐hundred and twenty adults with voice disorders were included in this study (Mean age = 53.6, SD = 16.5). Distribution of diagnoses was as follows: muscle tension dysphonia = 391 (32%), benign vocal fold lesions = 359 (29.4%), laryngospasm = 150 (12.3%), vocal fold paralysis or paresis = 135 (11.1%), neurological voice disorders = 76 (6.2%), laryngeal cancer or respiratory papilloma = 72 (5.9%), and presbyphonia = 37 (3%). Distribution of sex, GRBAS ratings, and ADI score across voice diagnoses is presented in Table 1. Overall, 429 patients were males (mean age = 56.0, SD = 16.6) and 788 were females (Mean age = 52.2, SD = 16.3). Twenty‐nine percent (*N* = 361) of patients reported having smoked in the past, and 4.9% (*N* = 60) were current smokers. Four‐hundred and ninety‐three participants reported fulltime employment (40.4%), 109 reported part time employment (8.9%), 297 were retired (24.3%), 48 reported being on disability (4%), and 29 indicated that they were unemployed (2.4%). Two‐hundred and forty‐four participants did not report their employment status. Regarding socioeconomic deprivation, one‐hundred and thirty‐six participants lived in areas scored 1–20 on the ADI (least socioeconomic deprivation), 557 in areas scored 21–40, 338 in areas scored 41–60, 138 in areas scored 61–80, and fifty‐one in areas scored 81–100 (greatest socioeconomic deprivation). No significant differences in age were observed between diagnostic (*p* = 0.91) or ADI groups (*p* = 0.73). Table 2 presents sex, smoking status, diagnosis, and GRBAS ratings across ADI groups.

**TABLE 1:** Distribution of sex, GRBAS ratings, and ADI score across voice diagnoses.

	Diagnosis							
	Muscle tension	Paralysis	Benign lesions	Neurological	Laryngospasm	Cancer	Presbyphonia	Total
Total *N* (% of diagnosis)	391	135	359	76	150	72	37	1220
GRBAS rating								
0	83 (21.2%)	23 (17.0%)	72 (20.1%)	15 (19.7%)	35 (23.3%)	13 (18.1%)	5 (13.5%)	246 (20.2%)
1	150 (38.4%)	60 (44.4%)	132 (36.8%)	28 (36.8%)	57 (38.0%)	22 (30.6%)	12 (32.4%)	461 (37.8%)
2	112 (28.6%)	44 (32.6%)	100 (27.9%)	25 (32.9%)	44 (29.3%)	27 (37.5%)	17 (45.9%)	369 (30.2%)
3	46 (11.8%)	8 (5.9%)	55 (15.3%)	8 (10.5%)	14 (9.3%)	10 (13.9%)	3 (8.1%)	144 (11.8%)
Area Deprivation Index								
0–20	44 (11.3%)	15 (11.1%)	47 (13.1%)	7 (9.2%)	16 (10.7%)	4 (5.6%)	3 (8.1%)	136 (11.1%)
21–40	188 (48.1%)	61 (45.2%)	149 (41.5%)	44 (57.9%)	69 (46.0%)	29 (40.3%)	17 (45.9%)	557 (45.7%)
41–60	94 (24.0%)	41 (30.4%)	111 (30.9%)	13 (17.1%)	41 (27.3%)	28 (38.9%)	10 (27.0%)	338 (27.7%)
61–80	49 (12.5%)	14 (10.4%)	39 (10.9%)	9 (11.8%)	16 (10.7%)	6 (8.3%)	5 (13.5%)	138 (11.3%)
81–100	16 (4.1%)	4 (3.0%)	13 (3.6%)	3 (3.9%)	8 (5.3%)	5 (6.9%)	2 (5.4%)	51 (4.2%)
Sex								
Male	126 (32.3%)	48 (35.6%)	124 (34.7%)	27 (35.5%)	60 (40.0%)	28 (38.9%)	16 (43.2%)	429 (35.3%)
Female	264 (67.7%)	87 (64.4%)	233 (65.3%)	49 (64.5%)	90 (60.0%)	44 (61.1%)	21 (56.8%)	788 (64.7%)

**TABLE 2:** Distribution of sex, smoking status, voice‐related diagnoses, and GRBAS ratings across ADI quintile groups.

	Area Deprivation Index					
	0–20	21–40	41–60	61–80	81–100	Total
Total *N* (% of quintile)	136 (100%)	557 (100%)	338 (100%)	138 (100%)	51 (100%)	1220 (100%)
Sex						
Males	58 (43%)	191 (34.4%)	108 (32%)	54 (39%)	18 (35.3%)	429 (35.3%)
Females	77 (57%)	365 (65.6%)	229 (68%)	84 (60.9%)	33 (64.7%)	788 (64.7%)
Smoking status						
Non‐smokers	90 (69.8%)	334 (65.5%)	170 (55.7%)	81 (64.3%)	19 (42.2%)	694 (62.2%)
Past smokers	36 (27.9%)	157 (30.8%)	114 (37.4%)	34 (27%)	20 (44.4%)	361 (32.4%)
Current smokers	3 (2.3%)	19 (3.7%)	21 (6.9%)	11 (8.7%)	6 (13.3%)	60 (5.4%)
Diagnosis						
Muscle tension	44 (32.4%)	188 (33.8%)	94 (27.8%)	49 (35.5%)	16 (31.4%)	391 (32%)
Paralysis	15 (11%)	61 (11%)	41 (12.1%)	14 (10.1%)	4 (7.8%)	135 (11.1%)
Benign lesions	47 (34.6%)	149 (26.8%)	111 (32.8%)	39 (28.3%)	13 (25.5%)	359 (29.4%)
Neurological	7 (5.1%)	44 (7.9%)	13 (3.8%)	9 (6.5%)	3 (5.9%)	76 (6.2%)
Laryngospasm	16 (11.8%)	69 (12.4%)	41 (12.1%)	16 (11.6%)	8 (15.7%)	150 (12.3%)
Cancer	4 (2.9%)	29 (5.2%)	28 (8.3%)	6 (4.3%)	5 (9.8%)	72 (5.9%)
Presbyphonia	3 (2.2%)	17 (3.1%)	10 (3%)	5 (3.6%)	2 (3.9%)	37 (3%)
GRBAS rating						
0	40 (29.4%)	124 (22.3%)	59 (17.5%)	19 (13.8%)	4 (7.8%)	246 (20.2%)
1	60 (44.1%)	212 (38.1%)	124 (36.7%)	56 (40.6%)	9 (17.6%)	461 (37.8%)
2	31 (22.8%)	157 (28.2%)	115 (34%)	46 (33.3%)	20 (39.2%)	369 (30.2%)
3	5 (3.7%)	64 (11.5%)	40 (11.8%)	17 (12.3%)	18 (35.3%)	144 (11.8%)

### 3.1 VHI Total Score

**FIGURE 1:**
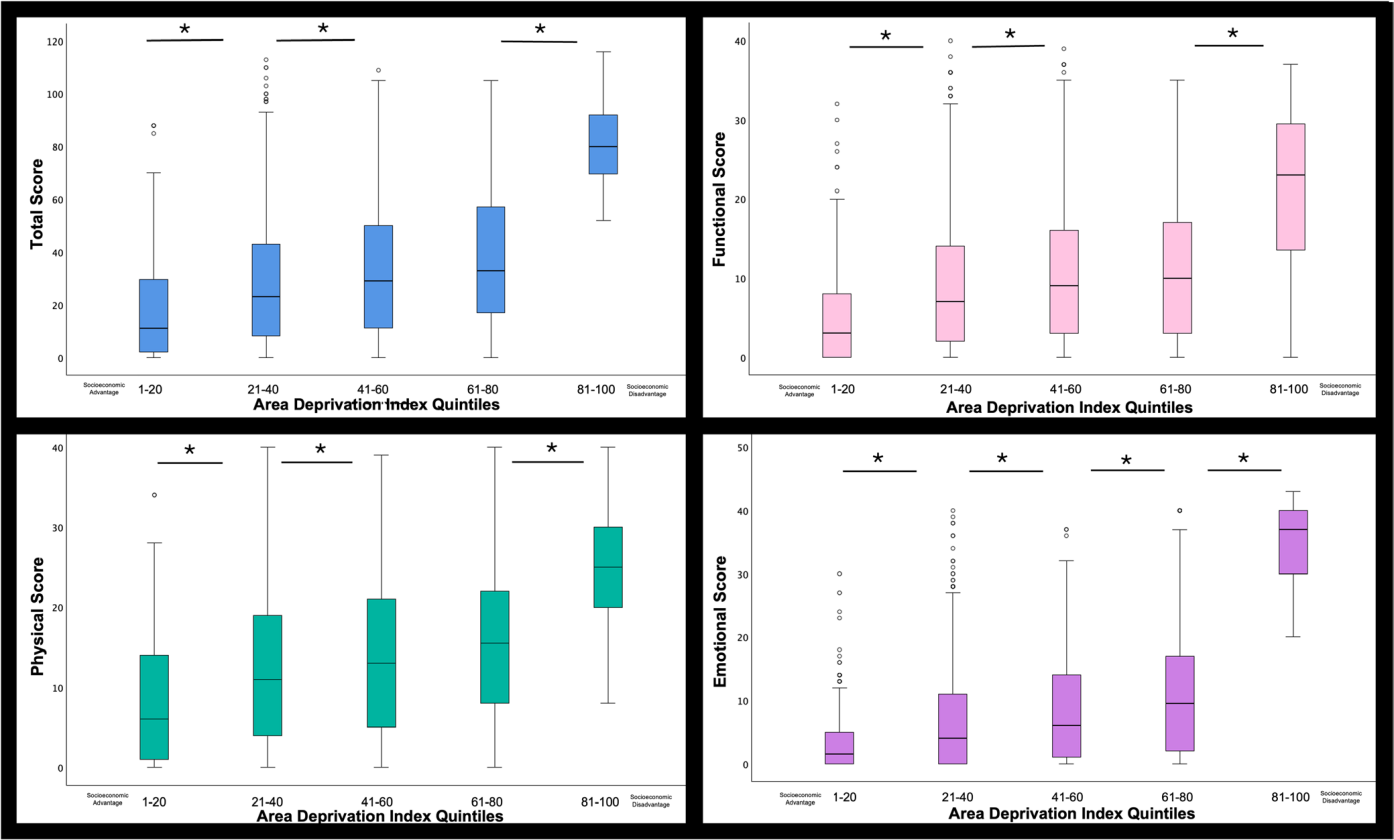
Boxplot for total, functional, physical, and emotional Voice Handicap Index (VHI) scores across patients living in Area Deprivation Index (ADI) quintiles. [Color figure can be viewed in the online issue, which is available at www.laryngoscope.com]

Mean VHI total and subscores across all the aforementioned factors are presented in Table 3. When controlling for the variables mentioned above, ADI and GRBAS scores were both significant predictors of VHI scores. Regarding socioeconomic deprivation, regression indicated a 0.32 increase in VHI score for every point increase in ADI score (*β* = 0.32, 95% CI = 0.26–1.26, *p* < 0.001). When comparing VHI scores across ADI quintiles, those living in areas 21–40 scored an average of 9.36 (95% CI = 4.9–13.7, *p* < 0.001) higher on the VHI than those living in areas scored 1–20 on the ADI (Figure 1). A mean increase of 4.54 (95% CI = 1.3–7.6, *p* < 0.001) was observed between groups 21–40 and 41–60, and a mean increase of 4.87 (95% CI = 0.26–9.4, *p* = 0.033) was observed between those in the 41–60 and 61–80 group. Finally, when comparing 61–80 and 81–100 groups, a mean increase of 42.7 was observed (95% CI = 35.2–50.2, *p* < 0.001). Complete statistical summary is presented in Supporting Information.

**TABLE 3:** Total, functional, physical, and emotional Voice Handicap Index (VHI) score means and standard deviations across Area Deprivation Index scores, sex, GRBAS ratings, voice‐related diagnoses, smoking history and employment status.

	Total score			Functional score			Physical score			Emotional score		
	Mean	SD	*N*	Mean	SD	*N*	Mean	SD	*N*	Mean	SD	*N*
Area Deprivation Index												
1–20	18.8	21.4	136	5.8	7.2	136	8.6	8.7	136	4.4	6.7	136
21–40	28.2	24.8	557	9.1	8.7	556	12.2	9.3	556	7.0	8.4	556
41–60	32.7	25.9	338	10.5	9.1	338	13.4	9.5	338	8.8	9.1	338
61–80	37.6	24.8	138	11.1	9.1	138	15.1	8.9	138	11.2	10.6	138
81–100	80.3	14.6	51	21.3	10.3	51	24.0	7.4	51	35.0	6.0	51
Sex												
Male	32.1	27.4	429	10.1	9.3	429	13.4	9.9	429	8.7	10.3	429
Female	31.4	26.6	788	9.7	9.2	787	12.7	9.4	787	8.9	10.4	787
GRBAS												
0 (Normal)	6.1	11.2	246	1.9	3.1	246	2.6	4.0	246	1.6	6.0	246
1 (Mild)	23.1	18.1	461	6.7	6.1	460	10.4	7.0	460	5.9	8.1	460
2 (Moderate)	43.9	21.4	369	13.7	7.6	369	18.3	7.0	369	12.1	9.5	369
3 (Severe)	71.1	19.7	144	23.8	8.0	144	24.9	6.9	144	22.4	8.9	144
Diagnosis												
Muscle tension	31.0	25.9	391	9.5	8.8	391	12.8	9.3	391	8.6	10.2	391
Paralysis	28.0	22.7	135	8.4	7.9	134	11.8	8.5	134	7.6	9.2	134
Benign lesions	32.8	28.3	359	10.4	9.8	359	13.4	10.1	359	9.2	10.5	359
Neurological	30.5	26.6	76	9.8	9.1	76	12.7	9.4	76	8.1	9.5	76
Laryngospasm	33.5	28.8	150	10.7	10.0	150	13.1	10.1	150	9.7	11.0	150
Cancer	32.1	29.7	72	9.7	10.0	72	13.2	10.2	72	9.2	11.3	72
Presbyphonia	33.1	23.9	37	9.6	8.4	37	13.1	7.8	37	10.3	11.4	37
Smoking history												
None	30.0	26.1	694	9.4	9.0	694	12.5	9.5	694	8.1	9.7	694
In the past	33.4	27.7	361	10.3	9.4	361	13.4	9.8	361	9.7	10.8	361
Current	33.7	29.2	60	10.2	9.9	60	13.0	9.8	60	10.5	13.1	60
Employment status												
Full time	31.3	26.8	493	9.7	9.2	492	12.8	9.7	492	8.7	10.2	492
Part time	26.9	24.8	109	8.2	8.2	109	11.6	9.5	109	7.2	9.4	109
Retired	31.1	27.2	297	9.5	9.3	297	12.8	9.7	297	8.8	10.4	297
Disabled	29.5	24.6	48	8.9	8.0	48	11.7	8.2	48	8.9	11.8	48
Unemployed	35.3	27.1	29	11.5	9.2	29	13.3	8.7	29	10.5	11.2	29

A significant relationship was also observed between GRBAS ratings and VHI total scores (Table 3). When compared with those rated as having normal voice quality (GRBAS = 0), those rated as having mild dysphonia reported total scores 15.1 points higher (*β* = 15.1, 95% CI = 12.1–18.1, *p* < 0.001), those with moderate dysphonia 35.6 points higher (*β* = 15.1, 95% CI = 12.1–18.1, *p* < 0.001), and those with severe dysphonia 59.8 points higher (*β* = 15.1, 95% CI = 12.1–18.1, *p* < 0.001). Neither sex, employment status, diagnosis, smoking history, nor age was significantly associated with total score.

### 3.2 Functional Score

Area Deprivation Index (ADI) scores also significantly predicted functional scores. Regression indicated a 0.06 increase in functional VHI score for every point increase in ADI score (*β* = 0.06, 95% CI = 0.04–0.08, *p* < 0.001). On average, VHI scores were an average of 3.31 points greater (95% CI = 1.6–4.9, *p* < 0.001) in those living in areas rated 21–40, when compared to those living in areas 1–20. A mean difference of 1.39 (95% CI = 0.2–2.5, *p* < 0.001) was observed between groups 21–40 and 41–60; however, no significant pattern was observed between those in the 41–60 and 61–80 groups. Scores were an average of 10.1 points higher for the 81–100 group when compared with the 61–80 group (95% CI = 7.2–12.9, *p* < 0.001; Figure 1).

A significant relationship was also observed between GRBAS ratings and functional scores (Table 3). Compared to those rated as having normal voice quality (GRBAS = 0), those rated as having mild dysphonia reported 4.42 points greater scores (*β* = 4.42, 95% CI = 3.3–5.4, *p* < 0.001), those with moderate dysphonia reported 11.5 points greater scores (*β* = 11.5, 95% CI = 10.4–12.7, *p* < 0.001), and those with severe dysphonia reported 20.5 points greater scores (*β* = 20.5, 95% CI = 19.0–22.0, *p* < 0.001). No other factors were significantly associated with functional score.

### 3.3 Physical Score

The ADI significantly predicted physical scores. Regression indicated a 0.08 increase in physical score for every point increase in ADI score (*β* = 0.08, 95% CI = 0.05–0.1, *p* < 0.001). When comparing those living in areas scored 1–20 on the ADI to those living in areas 21–40, VHI scores were an average of 3.56 points higher (95% CI = 1.9–5.21, *p* < 0.001). On average, scores were 1.24 points higher (95% CI = 0.04–2.4, *p* < 0.001) for those in the 41–60 group when compared to the 21–40 group. No significant difference was observed between those in the 41–60 and 61–80 groups. Finally, when comparing the 61–80 and 81–100 groups, a mean difference of 8.91 was observed (95% CI = 6.1–11.7, *p* < 0.001; Figure 1).

GRBAS ratings also predicted VHI physical score (Table 3). Compared to those rated as having normal voice quality (GRBAS = 0), those rated as having mild dysphonia reported scores 7.59 points greater (*β* = 7.59, 95% CI = 6.6–8.58, *p* < 0.001); those with moderate dysphonia reported 15.19 point larger scores (*β* = 15.19, 95% CI = 14.1–16.2, *p* < 0.001); and those with severe dysphonia reported 21.6 point greater scores (*β* = 21.6, 95% CI = 20.3–22.9, *p* < 0.001). No other factors were significantly associated with physical score.

### 3.4 Emotional Score

ADI scores also significantly predicted emotional scores. Regression indicated a 0.18 increase in emotional score for every point increase in ADI score (*β* = 0.18, 95% CI = 0.16–0.21, *p* < 0.001). When comparing those living in areas scored 1–20 on the ADI to those living in areas 21–40, VHI scores were greater by an average of 2.55 points (95% CI = 0.74–4.3, *p* < 0.001). A mean increase of 1.87 (95% CI = 0.56–3.1, *p* < 0.001) was observed between groups 21–40 and 41–60, and a mean difference of 2.4 (95% CI = 0.48–4.3, *p* < 0.001) was evident between those in the 41–60 and 61–80 group. Finally, when comparing the 61–80 and 81–100 groups, scores were an average of 23.7 points higher for those in the 81–100 group (95% CI = 20.6–26.8, *p* < 0.001; Figure 1).

GRBAS ratings also predicted VHI emotional score (Table 3). Compared to those rated as having normal voice quality (GRBAS = 0), those rated as having mild dysphonia reported 3.5 point greater scores (*β* = 3.5, 95% CI = 2.1–4.8, *p* < 0.001), those with moderate dysphonia 8.95 point higher scores (*β* = 8.95, 95% CI = 7.55–10.35, *p* < 0.001), and those with severe dysphonia 18.37 point larger scores (β = 18.37, 95% CI = 16.56–20.18, *p* < 0.001). No other factors were significantly associated with emotional score.

## 4 Discussion

Voice disorders impede communication and carry social and emotional ramifications [48, 49]. Measures such as the VHI are designed to quantify the extent to which an individual's voice problems disrupt their quality of life [18]. Little is known, however, regarding the manner in which socioeconomic deprivation influences voice‐related handicap. This study examined the relationship between the VHI and socioeconomic deprivation as measured by the ADI in adults with voice disorders. It was hypothesized that individuals living in areas experiencing higher levels of socioeconomic deprivation would experience greater voice‐related handicap in daily life. This hypothesis was confirmed as patients from the least affluent neighborhoods presented with significantly greater VHI scores, even after controlling for severity of dysphonia, employment status, age, sex, smoking status, and voice‐related diagnosis. This suggests that patients living with socioeconomic deprivation may experience voice disorders differently than more affluent individuals.

There are several factors that might exacerbate the social and emotional impact of voice disorders for individuals living in disadvantaged areas. One factor might be the timing of and access to assessment and treatment for voice disorders. Patients from lower income homes have been documented to postpone treatment for longer periods of time following symptom onset [32]. These individuals may delay treatment because specialist providers are difficult to access [31, 50]. As a result, the patients in this study may have been experiencing symptoms for some time before they were followed for treatment, and the delay may have increased the negative impact of their voice problems. In addition, there may be economic, schedule‐related, and geographical barriers to treatment for individuals in disadvantaged areas [31, 51].

As another potential factor, public awareness of otolaryngology‐related treatment options is often limited [52], and many individuals may not realize how or where their symptoms can be addressed. This might explain why only 4.5% of patients in the current study came from neighborhoods experiencing the greatest levels of socioeconomic deprivation. The treatment‐seeking population for voice disorders has typically been White, educated, and relatively affluent [53], and this may reflect a disparity of care associated with economic deprivation. This finding suggests that enhanced outreach and increased treatment options are needed to provide equitable care for patients across the social and economic continuum.

A number of environmental factors associated with socioeconomic deprivation could disrupt vocal function and thereby limit quality of life. For example, lower income neighborhoods often experience higher levels of pollution and airborne irritants [54, 55]. The relationship between airborne irritants and voice disorders is unclear; however, nanoparticle exposure has been observed to induce a fibrotic phenotype in vocal fold fibroblasts [56]. Additionally, acrolein exposure (a pollutant found in exhaust, smoke, and industrial waste) can impair vocal fold epithelial barrier integrity and cause edema in animal models [57, 58]. Collectively, these findings suggest that pollution may adversely influence vocal function [59] and prevent spontaneous resolution of voice symptoms.

As another consideration, socioeconomic status is associated with greater levels of noise pollution [60]. Past study suggests that the influence of noise pollution, particularly in occupational settings, can negatively influence vocal health [61]. For example, high levels of phonation at elevated vocal intensity have been observed in individuals with phonotraumatic vocal fold lesions [62]. Even in those with healthy vocal folds, communicating in ambient noise has induced vocal fatigue symptoms relatively quickly [63, 64, 65, 66]. These symptoms may accumulate over time, aggravate voice symptoms, result in voice disorders, and affect participation in many social and occupational activities [67].

It was interesting to note that although all VHI subscores displayed a significant association with the ADI, this relationship was strongest for Total and Emotional subscores. This finding suggests that emotional factors drove increased voice‐related handicap in those from poorer areas. The Emotional section of the VHI probes the presence of negative emotions such as embarrassment, annoyance, and shame [18]. Patients from less affluent neighborhoods may have experienced these emotions as if they felt less comfortable in medical settings and/or experienced greater levels of discrimination from providers [68]. This has, in fact, been postulated to be the reason why patients from lower SES use a more limited vocal pitch and intensity range in formal research/medical settings [69]. The Emotional section of the VHI also probes lost income associated with voice problems, which may have been a greater obstacle for patients experiencing socioeconomic deprivation as jobs in low SES areas may be less accommodating for illness.

It is unclear what constitutes a clinically significant change on the VHI. On the VHI‐10, minimal important difference is thought to be 6 points [70]. If this holds for the VHI, a 19‐point change in ADI score would be associated with a meaningful change in total VHI score. This aligns with past study suggesting those living in the most disadvantaged areas (highest quartile or greater) of the ADI display the poorest patient‐reported outcome measures [22, 71, 72]. For example, following hip arthroscopy, patients with ADI scores > 75 (greatest socioeconomic deprivation) report worse pain scores [71]. Similarly, cancer patients from high ADI areas report greater levels of anxiety [72]. Future work is needed to fully understand the relationship between the VHI and patient‐reported outcomes.

Several factors might be considered when interpreting the current data. First, the study population was racially homogenous, so race could not be considered in the regression model. Future studies should examine the relationship between race and SES as these factors are frequently related [73]. Additionally, prospective, multisite investigations should extend this line of work to examine the influence of SES on voice treatment outcomes—particularly as socioeconomic deprivation has been shown to influence cost and treatment outcomes of other upper airway conditions [74, 75]. Although the ADI provides an effective method of quantifying socioeconomic deprivation, measuring SES on a neighborhood level is not a perfect methodology. Thus, future broader‐based investigations employing a variety of SES metrics are warranted to confirm and extend the current findings. Additionally, the effect of employment status should be further studied as data were unavailable for some of the current population.

## 5 Conclusions

Patients with voice disorders living in areas with greater socioeconomic deprivation reported greater voice‐related handicap than those living in more affluent areas. This effect was observed even when controlling for dysphonia severity, age, sex, voice‐related diagnosis, employment status, and smoking history. The association between socioeconomic deprivation and VHI scores was strongest for Total and Emotional subscores, suggesting that individuals living with greater socioeconomic deprivation may experience more emotional sequalae from voice disorders than those from more affluent backgrounds. Future work is needed to further characterize the relationship between voice disorders and SES, identify the mechanisms underlying these associations, and promote equitable care for individuals across the social and economic spectrum.

## Funding

This work was supported by National Institute on Deafness and Other Communication Disorders, T32‐DC009401.

## Conflicts of Interest

The authors declare no conflicts of interest.

## Supporting information


**Supporting Information.** Statistical summary for regression models examining total, functional, physical and emotional Voice Handicap Index subscores.

## Data Availability

The data that support the findings of this study are available on request from the corresponding author. The data are not publicly available due to privacy or ethical restrictions.
