# Supplementary material for: Socioeconomic Deprivation and Vocal Handicap in Adults With Voice Disorders
Source: Laryngoscope. 2026 Mar 23;136(8):3503–13. doi: 10.1002/lary.70510 (PMC13036882; doi:10.1002/lary.70510)
Supplement: Supplementary file 1 — Supporting Information. Statistical summary for regression models examining total, functional, physical and emotional Voice Handicap Index subscores. [file LARY-136-3503-s001.docx]

Supporting Information: Statistical Summary

**Total Voice Handicap Index (VHI) Score**

Var: ADI + GRBAS + age + sex + employment status + smoking + diagnosis

|  | effect | 2.5 % | 97.5 % | p.val |
| --- | --- | --- | --- | --- |
| (Intercept) | -4.16 | -9.96 | 1.63 | 0.16 |
| ADI | 0.32 | 0.26 | 0.38 | <0.001 |
| GRBAS - 1 | 15.11 | 12.15 | 18.08 | <0.001 |
| GRBAS - 2 | 35.68 | 32.51 | 38.86 | <0.001 |
| GRBAS - 3 | 59.89 | 55.78 | 64 | <0.001 |
| Age | -0.02 | -0.11 | 0.07 | 0.672 |
| Sex | 0.26 | -2.1 | 2.61 | 0.832 |
| Employment (parttime) | -1.34 | -5.05 | 2.38 | 0.481 |
| Employment (retired) | 0.75 | -2.6 | 4.1 | 0.662 |
| Employment (disabled) | -3.72 | -9.39 | 1.96 | 0.2 |
| Employment (unemployed) | 5.93 | -7.03 | 18.89 | 0.37 |
| Employment (parttime) | 5.19 | -1.38 | 11.77 | 0.122 |
| Past smoker | 1.31 | -1.18 | 3.79 | 0.302 |
| Current smoker | 1 | -3.94 | 5.94 | 0.691 |
| Paralysis | -3.47 | -7.32 | 0.39 | 0.078 |
| Benign lesions | 0.29 | -2.49 | 3.08 | 0.836 |
| Neurological | -1.86 | -6.53 | 2.81 | 0.435 |
| Laryngospasm | 1.22 | -2.52 | 4.96 | 0.523 |
| Cancer/RPP | -3.3 | -8.32 | 1.72 | 0.197 |
| Presbyphonia | -2.53 | -9.57 | 4.5 | 0.481 |

Functional VHI Score

Var: ADI + GRBAS + age + sex + employment status + smoking + diagnosis

|  | effect | 2.5 % | 97.5 % | p.val |
| --- | --- | --- | --- | --- |
| (Intercept) | 0.83 | -1.27 | 2.92 | 0.441 |
| ADI | 0.06 | 0.04 | 0.08 | <0.001 |
| GRBAS - 1 | 4.42 | 3.35 | 5.49 | <0.001 |
| GRBAS - 2 | 11.56 | 10.41 | 12.71 | <0.001 |
| GRBAS - 3 | 20.53 | 19.04 | 22.02 | <0.001 |
| Age | -0.02 | -0.05 | 0.01 | 0.212 |
| Sex | -0.34 | -1.2 | 0.51 | 0.428 |
| Employment (parttime) | -0.31 | -1.66 | 1.03 | 0.648 |
| Employment (retired) | 0.49 | -0.73 | 1.7 | 0.431 |
| Employment (disabled) | -1.26 | -3.31 | 0.8 | 0.231 |
| Employment (unemployed) | 3.17 | -1.53 | 7.86 | 0.186 |
| Employment (parttime) | 2.45 | 0.07 | 4.83 | 0.044 |
| Past smoker | 0.41 | -0.49 | 1.31 | 0.374 |
| Current smoker | 0.39 | -1.39 | 2.18 | 0.666 |
| Paralysis | -1.07 | -2.48 | 0.33 | 0.133 |
| Benign lesions | 0.25 | -0.76 | 1.26 | 0.63 |
| Neurological | 0.14 | -1.56 | 1.83 | 0.875 |
| Laryngospasm | 1.1 | -0.26 | 2.45 | 0.113 |
| Cancer/RPP | -1.08 | -2.89 | 0.74 | 0.246 |
| Presbyphonia | -1.44 | -3.99 | 1.11 | 0.268 |

Physical VHI Score

Var: ADI + GRBAS + age + sex + employment status + smoking + diagnosis

|  | effect | 2.5 % | 97.5 % | p.val |
| --- | --- | --- | --- | --- |
| (Intercept) | 0.98 | -1.15 | 3.11 | 0.367 |
| ADI | 0.08 | 0.05 | 0.1 | <0.001 |
| GRBAS - 1 | 7.13 | 6.04 | 8.21 | <0.001 |
| GRBAS - 2 | 15.34 | 14.17 | 16.5 | <0.001 |
| GRBAS - 3 | 21 | 19.49 | 22.51 | <0.001 |
| Age | -0.01 | -0.04 | 0.02 | 0.505 |
| Sex | -0.29 | -1.15 | 0.58 | 0.517 |
| Employment (parttime) | 0.11 | -1.25 | 1.48 | 0.874 |
| Employment (retired) | 0.48 | -0.75 | 1.71 | 0.445 |
| Employment (disabled) | -1.2 | -3.28 | 0.88 | 0.259 |
| Employment (unemployed) | -1.74 | -6.5 | 3.02 | 0.473 |
| Employment (parttime) | 0.77 | -1.65 | 3.18 | 0.533 |
| Past smoker | 0 | -0.92 | 0.91 | 0.995 |
| Current smoker | -0.02 | -1.83 | 1.8 | 0.985 |
| Paralysis | -1.54 | -2.97 | -0.12 | 0.033 |
| Benign lesions | 0.15 | -0.87 | 1.17 | 0.772 |
| Neurological | -1.13 | -2.85 | 0.58 | 0.196 |
| Laryngospasm | -0.09 | -1.47 | 1.28 | 0.894 |
| Cancer/RPP | -0.96 | -2.8 | 0.89 | 0.31 |
| Presbyphonia | -1.68 | -4.27 | 0.9 | 0.202 |

Emotional VHI Score

Var: ADI + GRBAS + age + sex + employment status + smoking + diagnosis

|  | effect | 2.5 % | 97.5 % | p.val |
| --- | --- | --- | --- | --- |
| (Intercept) | -5.82 | -8.37 | -3.26 | <0.001 |
| ADI | 0.18 | 0.16 | 0.21 | <0.001 |
| GRBAS - 1 | 3.5 | 2.19 | 4.8 | <0.001 |
| GRBAS - 2 | 8.95 | 7.55 | 10.35 | <0.001 |
| GRBAS - 3 | 18.37 | 16.56 | 20.18 | <0.001 |
| Age | 0.01 | -0.03 | 0.05 | 0.588 |
| Sex | 0.75 | -0.29 | 1.79 | 0.157 |
| Employment (parttime) | -1.04 | -2.68 | 0.6 | 0.213 |
| Employment (retired) | -0.03 | -1.51 | 1.45 | 0.97 |
| Employment (disabled) | -1.18 | -3.68 | 1.32 | 0.354 |
| Employment (unemployed) | 4.64 | -1.07 | 10.36 | 0.111 |
| Employment (parttime) | 2.04 | -0.86 | 4.94 | 0.168 |
| Past smoker | 0.8 | -0.3 | 1.89 | 0.154 |
| Current smoker | 0.66 | -1.52 | 2.83 | 0.554 |
| Paralysis | -1.24 | -2.94 | 0.47 | 0.155 |
| Benign lesions | 0.06 | -1.16 | 1.29 | 0.918 |
| Neurological | -0.82 | -2.88 | 1.23 | 0.433 |
| Laryngospasm | 0.21 | -1.44 | 1.86 | 0.803 |
| Cancer/RPP | -1.36 | -3.57 | 0.85 | 0.229 |
| Presbyphonia | 0.53 | -2.57 | 3.63 | 0.736 |
